# Large-Scale “OMICS” Studies to Explore the Physiopatholgy of HIV-1 Infection

**DOI:** 10.3389/fgene.2019.00799

**Published:** 2019-09-13

**Authors:** Sigrid Le Clerc, Sophie Limou, Jean-François Zagury

**Affiliations:** ^1^Laboratoire GBCM, EA7528, Conservatoire National des Arts et Métiers, HESAM Université, Paris, France; ^2^Centre de Recherche en Transplantation et Immunologie UMR1064, INSERM, Université de Nantes, Nantes, France; ^3^Institut de Transplantation en Urologie et Néphrologie (ITUN), CHU de Nantes, Nantes, France; ^4^Computer Sciences and Mathematics Department, Ecole Centrale de Nantes, Nantes, France

**Keywords:** HIV, genome-wide association study, genomics, omics, big data

## Abstract

In this review, we present the main large-scale experimental studies that have been performed in the HIV/AIDS field. These “omics” studies are based on several technologies including genotyping, RNA interference, and transcriptome or epigenome analysis. Due to the direct connection with disease evolution, there has been a large focus on genotyping cohorts of well-characterized patients through genome-wide association studies (GWASs), but there have also been several *invitro* studies such as small interfering RNA (siRNA) interference or transcriptome analyses of HIV-1–infected cells. After describing the major results obtained with these omics technologies—including some with a high relevance for HIV-1 treatment—we discuss the next steps that the community needs to embrace in order to derive new actionable therapeutic or diagnostic targets. Only integrative approaches that combine all big data results and consider their complex interactions will allow us to capture the global picture of HIV molecular pathogenesis. This novel challenge will require large collaborative efforts and represents a huge open field for innovative bioinformatics approaches.

## Introduction

HIV remains a major global public health issue, having claimed more than 35 million lives so far. In 2017, 940,000 people died from HIV-related causes globally (Global AIDS Update, 2016). The active anti-retroviral therapies are efficient and have saved many lives but still present multiple caveats: need for high compliance, permanent treatment, and unwanted side effects and complications (Li et al., 2017). Developing alternative and simple solutions such as immunoprophylactic or immunotherapeutic options remains a public health priority. In line with this, a better understanding of the molecular etiology of disease progression is essential.

Due to the impact of the disease in the Western world, HIV research has been the subject of intense efforts for the past 35 years and has helped in promoting several new research technologies, in particular with high-throughput studies from the “omics” era.

In this review, we will present large-scale studies based on various technologies that have been undertaken to tackle HIV molecular etiology and their main results. These large-scale studies encompass mainly genomics with genome-wide association studies (GWASs) based on genotyping chips and with exome sequencing, transcriptomic studies from SIV and HIV patient cells, and small interfering RNA (siRNA) studies in sensitive cell lines. We will also briefly describe the results obtained from proteomics and epigenomics screenings.

## A Short Recall on HIV-1 Infection and Markers

There are three successive stages in HIV infection: the acute primary infection, the asymptomatic stage and symptomatic HIV infection, and acquired immunodeficiency syndrome (AIDS). Depending on the individual, AIDS, the most advanced stage of the infection course, can occur within a few months to several years after HIV infection, with an average of around 8 years in the Western world. This stage has been defined by the Center for Disease Control (CDC) either as a drop of CD4 T-cell count below 200/mm^3^ or as the appearance of opportunistic infections or some cancers (Center for Disease Control and Prevention, 1992). Quite early, AIDS cohorts were enrolled and prospectively followed, and it became apparent that this infection was exhibiting a considerable phenotypic heterogeneity at different levels: virus acquisition, disease progression, viral load control, and response to treatment (Langlade-Demoyen et al., 1994; Ludlam et al., 1985; Fowke et al., 1996; Pantaleo and Fauci, 1996; Hetherington et al., 2002; Mallal et al., 2002; Saksena et al., 2007). For instance, some individuals, called long-term non-progressors, are infected but never progress to AIDS; the elite controllers have never exhibited any detectable viral load; and the rapid progressors reach the AIDS stage within a few months following their infection. This phenotypic variability may be attributed to a complex interplay between viral, environmental, and host genetic factors that could be investigated through several types of large-scale studies or omics.

If the CD4 cell count was the major marker to follow HIV-1 infection and immune deficiency in patients in the early 1980s, the progress of molecular biology techniques has made it possible to measure precisely HIV-1 viral load in the blood (i.e., the number of viral particles present in each ml of serum) by the late 1990s. Together with the CD4 cell count, this marker has become very useful to evaluate the status of an infected patient, either a low viral load suggesting a good control of HIV-1 infection or a high viral load suggesting a progressive infection at an early stage of infection or an uncontrolled infection at a late disease stage (AIDS). Most cohorts focus on viral load outcomes (e.g., viral control, viral load at set point), but (slow and rapid) progression phenotypes have also been defined based on the CD4 counts (e.g., the GRIV cohort).

## Genetic Association Studies

Host genes associated with various phenotypes have been extensively explored since the mid-1990s. The concept is as follows: if a particular phenotype (for instance, elite control) is statistically associated with the presence or absence of a genetic variant, the corresponding gene or its product may be involved in the molecular mechanisms of viral infection/dissemination. Genetic association studies can thus provide new clues on the molecular mechanisms of infection and disease progression and, in the long run, identify new targets for the development of new therapeutic or diagnostic strategies. Initial studies have focused on candidate genes such as *HLA* (Kaslow et al., 1996; Carrington et al., 1999; Hendel et al., 1999), and a large number of host genetic associations with HIV outcomes have been identified. The main confirmed association was a 32-base-pair deletion in the *CCR5* gene (*CCR5-Δ32*) (Dean et al., 1996; Liu et al., 1996; Samson et al., 1996; Rappaport et al., 1997), but other variants closely located in the *CCR5* promoter (Martin et al., 1998; McDermott et al., 1998) or in the nearby *CCR2* gene (Smith et al., 1997) were also influential. This deletion led to the expression of a truncated and non-functional cell surface CCR5 protein that happened to be the major HIV-1 entry co-receptor (Alkhatib et al., 1996; Deng et al., 1996; Liu et al., 1996). Other candidate gene studies pointed to immunity-related genes (e.g., *KIR*, *IL10*, *IFNγ*) and genes encoding HIV restriction factors (e.g., *CCL5*, *APOBEC3G*, *CUL5*) but were only partially replicated according to the phenotype and cohort tested. The functional interpretation for most of these variants is yet to be discovered, but the detailed account of each candidate gene is beyond the scope of this article and has been covered by previous enlightening reviews (Fellay, 2009; An and Winkler, 2010). Overall, most of the candidate gene associations displayed small to modest effect sizes and, combined all together, account for a small fraction of the phenotype variability (O’Brien and Nelson, 2004).

## Genome-Wide Association Studies

It was only in 2007 that the first large-scale genetic association studies or GWASs have been published with the seminal publication by Fellay et al. that focused mainly on viral load at set point as the main phenotype of interest (Fellay et al., 2007). These large-scale genomic studies have relied on genotyping chips targeting simultaneously hundreds of thousands to millions of specific genetic markers called single nucleotide polymorphisms (SNPs), the most frequent polymorphisms in the human genome, that can be rapidly and easily genotyped. In contrast to the candidate gene strategy, this approach measures and analyzes gene variants across the whole human genome in an effort to identify common genetic risk factors in the population without any biological hypothesis *apriori*. Since the 2007 publication, GWASs have taken the place of candidate gene studies in AIDS. More than 20 GWASs focusing on various phenotypes and cohorts have been published, and **Table 1** summarizes these studies with their main characteristics (see also (Limou and Zagury, 2013)): date of the publication, origin of the cohort, size of the cohort, phenotype(s) of interest, genotyping chip type, associated SNP(s), best P-value, possible causing gene(s) involved, and publication reference number.

**Table 1:** GWASs published in the AIDS field since 2007. 5×10^−8^ is the standard threshold for considering an association as significant in a GWAS, and the significant ones in the table are in bold.

Publication year	Discovery sample origin	Discovery sample size	Reported trait	Microarray type	Main SNPs	P-value	Gene(s)	Reference
2007	European	486	HIV-1 viral set point	Illumina HumanHap550	rs239502, rs9264942	**9.36×10^−12^**, **3.77×10^−9^**	HLA-B*5701	(Fellay et al., 2007)
2009	European	2362	HIV-1 control (elite and viremic)	Illumina HumanHap550, Illumina Human1M	rs239502, rs9264942	**5×10^−35^**, **6×10^−32^**	HCP5/HLA-B*5701, *HLA-C*	(Fellay et al., 2009)
2009	European	1071	AIDS progression	Illumina HumanHap550, Illumina Human1M	rs9264942, rs2395029	**6×10^−12^**, **1×10^−11^**	*HLA-C*, HCP5/HLA-B*5701	(Fellay et al., 2009)
2009	European	85 rapid progressors HIV(+), 1,352 CTR HIV(−)	AIDS progression	Illumina HumanHap300	rs4118325	6×10^−7^	*PRMT6*	(Le Clerc et al., 2009)
2009	European	275 non-progressors HIV(+), 1,352 CTR HIV(−)	AIDS progression	Illumina HumanHap300	rs2395029	**6.79×10^−10^**, **6.79×10^−8^**	HCP5/HLA-B*5701	(Limou et al., 2009)
2010	European	156	HIV-1 progression	Affymetrix Human Mapping 500K	rs17762192	6.23×10^−7^	*PROX1-AS1*	(Herbeck et al., 2010)
2010	African American or Afro-Caribbean	515	HIV-1 viral set point	Illumina HumanHap1M, HumanHap1M-Duo, HumanHap550K	rs454422	1.46×10^−6^	*MCM8*	(Pelak et al., 2010)
2010	European	1712	HIV-1 control	Illumina HumanHap650Y	rs9264942, rs4418214	**6.3×10^−16^**	*HLA-C*, *MICA*	(International H.I.V. Controllers Study et al., 2010)
2010	African American or Afro-Caribbean	677	HIV-1 control	Illumina HumanHap650Y	rs2523608	**3.7×10^−15^**	*-*	(International H.I.V. Controllers Study et al., 2010)
2010	Sub-Saharan African	848 high-risk HIV(−), 531 HIV(+)	HIV-1 susceptibility	Illumina Human1M or 1M-Duo	rs842304	3.97×10^−6^	*-*	(Petrovski et al., 2011)
2010	Sub-Saharan African	100 HIV(+) infants, 126 HIV(−)	HIV susceptibility (mother-to-child transmission)	Illumina HumanHap650Y	rs12306	3.92×10^−5^	*WSB1*	(Joubert et al., 2010)
2011	European	191	HIV-1 replication *invitro*	Illumina Human610-Quad	rs12483205	2.16×10^−5^	*DYRK1A*	(Bol et al., 2011)
2011	Sub-Saharan African, African unspecified	302 high-risk HIV(−), 496 HIV(+)	HIV-1 susceptibility	Illumina HumanHap1M-Duo	rs3745760	7.83×10^−7^	*GLTSCR1*	(Lingappa et al., 2011)
2011	Sub-Saharan African, African unspecified	403	HIV-1 viral set point	Illumina HumanHap1M-Duo	rs13111989	2.15×10^−7^	*VEGFC*	(Lingappa et al., 2011)
2011	European	755	AIDS progression	Affymetrix Human Array 6.0	rs11884476	**3.37×10^−9^**	*PARD3B*	(Troyer et al., 2011)
2011	European	404	HIV-1 progression	Illumina HumanHap300	rs1523635	3.5×10^−6^	*AGR3*	(van Manen et al., 2011)
2013	European	430 high-risk HIV(−) and 765 HIV(+)	HIV-1 susceptibility	Illumina Human1M or 1M-Duo	-	-	*-*	(Lane et al., 2013)
2013	European	6,334 HIV-1(+), 7,247 HIV-1(−)	HIV-1 susceptibility	Meta-analysis/imputation	-	-	*-*	(McLaren et al., 2013)
2015	African American or Afro-Caribbean and European	628 high-risk HIV(−) vs. 1,376 HIV(+), 327 high-risk HIV(−) vs. 805 HIV(+)	HIV-1 susceptibility	Illumina HumanOmni1-Quad	rs4878712	4.38×10^−8^, 7.78×10^−4^	*CD33*, *FRMPD1*	(Johnson et al., 2015)
2015	European	6,315	HIV-1 viral set point	Meta-analysis/imputation	rs59440261, rs1015164	**2×10^−83^**, **1.5×10^−19^**	HLA-B*5701, *CCRL2*	(McLaren et al., 2015)
2015	East Asian	538	HIV-1 viral set point	Illumina Human660W-Quad	rs2442719	8×10^−7^	*HLA-B*	(Wei et al., 2015)
2017	Sub-Saharan Africans	556	AIDS progression	Illumina HumanOmni2.5M	rs2535307	3.72×10^−7^	*HCG22*	(Xie et al., 2017)

CTR, controls

The following major conclusions can be underlined from these GWA studies:

1. One signal was repeatedly replicated in several cohorts (of European descent) both for the viral load phenotype and also for the non-progression phenotype: rs2395029, an SNP in the *HCP5* gene within the *MHC* region, which was described in nearly full linkage disequilibrium (LD) with the *HLA*-B*5701 allele (Fellay et al., 2007; Dalmasso et al., 2008; Limou et al., 2009; McLaren et al., 2013; McLaren et al., 2017). This *HLA* allele could be critical for the destruction of infected cells *via* a yet-unidentified CD8 T-cell epitope, but we cannot exclude a role for other polymorphisms in the *HLA* region and possibly outside of the class I *HLA* genes, with relevant genes highlighted through LD such as *MICB* and *TNF* (Kulkarni et al., 2011). Similarly, in African Americans, the *HLA*-B*5703 allele was demonstrated to be important for viral control (Pelak et al., 2010; McLaren et al., 2012; McLaren et al., 2017). Beyond B*57 alleles, *HLA* seems to play a major role in viral control, as another SNP located 35 kb upstream of *HLA-C* was also identified in Europeans (Limou et al., 2009; McLaren et al., 2017). This −35 kb SNP was correlated with higher HLA-C cell surface expression (Thomas et al., 2009), which can be regulated through microRNA (miRNA) binding (Kulkarni et al., 2011) and through the binding of the Oct1 transcription factor (Vince et al., 2016).
2. In 2011, the HIV/AIDS research community conducted a massive effort to bring together their genomic data in order to gain statistical power of detection. This collaborative effort, named the International Consortium for the Genetics of HIV-1 (ICGH), gathered up to 6,300 HIV-infected individuals and 7,200 HIV-uninfected controls who were genotyped on various Illumina or Affymetrix platforms. A first publication emerged from this consortium in 2013; it focused on HIV-1 acquisition and confirmed the major role of *CCR5*-Delta32 in resistance to infection but did not identify any additional common genetic variant (McLaren et al., 2013). A second study was published 2 years later on viral control and disease progression and confirmed the complementary roles of *CCR5* locus variants and of *HLA* genetic variants coding for amino acids in the peptide binding grooves of HLA-A and HLA-B (McLaren et al., 2015). In spite of the large sample size, no other locus reached genome-wide significance. These reports conclude that future genetic studies should target other classes of genetic variants (e.g., low or rare frequency), non-European populations, and well-defined homogeneous phenotypes (McLaren et al., 2015).
3. In addition, the numerous GWASs have identified novel candidate genes, which all deserve further exploration, notably the ones that reached genome-wide significance (see **Table 1**). In particular, a genetic variant within the *CXCR6* gene was associated with long-term non-progression in four independent cohorts (Limou et al., 2010). This signal was only identified in individuals without sustained viral control, which explains why it was not highlighted in the consortium that mainly focused on viral control. Finally, the absence of replication for many other signals presented in **Table 1** does not discount their scientific interest but complicates their biological interpretation.

## Next-Generation Sequencing Studies

GWASs rely on common SNPs (typically >1% in the population) and hardly take into account the possible effect of rare variants or other classes of genetic polymorphisms such as indels or copy number variants that can also significantly impact disease outcome. To investigate the impact of such variants, we first focused on low-frequency SNPs (<5%) in our progression GWASs and identified the gene *RICH2* associated with non-progression, which interacts with BST-2, a major known HIV restriction factor (Le Clerc et al., 2011). Later, several studies based on next-generation sequencing (NGS) have emerged. Due to the high cost of such studies and to maximize statistical power of detection, these screenings have targeted coding variants (exome) and patients with very specific and extreme disease outcome. To our knowledge, only one publication has emerged from these studies (McLaren et al., 2017), which focused on 1,327 subjects, many of whom were elite and viral controllers. In spite of the significant number of patients studied, only variants in the *HLA* region came out, and this study suggested that exonic variants with large effect sizes are unlikely to have a major contribution to host control of HIV infection (McLaren et al., 2017).

## Functional Genomic Screenings

Systematic inactivation of gene expression through siRNA and small hairpin RNA (shRNA) offers a unique chance to identify host genes required for HIV replication. In large-scale studies, authors used siRNAs (Brass et al., 2008; Konig et al., 2008; Zhou et al., 2008) or shRNAs (Yeung et al., 2009) to silence *invitro* most known genes one by one in HIV permissive cell lines. These screenings identified 273 (Brass et al., 2008), 213 (Konig et al., 2008), 311 (Zhou et al., 2008), and 252 (Yeung et al., 2009) HIV host dependency factors, respectively, for a total of 842 putative candidates. However, the overlap between the different studies was very low, suggesting low reproducibility and/or high false positive, which might not be surprising considering the different experimental models (cell lines, HIV strains, and measurement modes of HIV replication). Overall, these studies still provide an interesting list of candidate cellular factors and pathways potentially implicated in HIV-1 replication that could be considered as relevant targets for drug development.

The development of the CRISPR–Cas9 technology to screen each gene with a library of single-guided RNA offers a greater sensitivity and specificity than interference based-RNA (Wang et al., 2014). A recent report used this technology to screen a CD4 T-cell line and identified five host factors required for HIV replication, including CD4, C-C motif chemokine receptor 5 (CCR5), and activated leukocyte cell adhesion molecule (ALCAM (Park et al., 2017). These factors were further validated in primary human CD4 T-cells and therefore represent major candidates for a therapeutic intervention.

## Transcriptomic Studies

The first descriptions of transcriptome analysis by DNA microarrays were in cancer in 2002 (Pomeroy et al., 2002; van ‘t Veer et al., 2002). In AIDS, the first large-scale transcriptomic study (4,600 transcripts) was published in 2003 (van ‘t Wout et al., 2003). This study analyzed gene expression in HIV-infected CD4 T-cell lines at different time points and revealed the inhibition of genes involved in cell division, transcription, translation, splicing, and also cholesterol biosynthesis (van ‘t Wout et al., 2003). An exon transcriptome microarray analysis of purified HIV-infected cells revealed host cell factors required for viral replication and alternative splicing events (Imbeault et al., 2012). A bioinformatic analysis of HIV-resistant activated CD4 T-cells (due to CD3/CD28 antibodies’ co-stimulation) highlighted a few dozen genes critical for resistance or permissivity (Xu et al., 2013).

Several microarray studies focused on non-human primate models, such as cynomolgus monkeys (Bosinger et al., 2004) and African green monkeys (non-pathogenic model) vs. rhesus macaques (pathogenic model) (Jacquelin et al., 2009). These reports mainly identified a major role for IFN-stimulated genes, as well as a differential expression of some innate genes (such as LPS receptors CD14 and TLR4) and some apoptosis-related genes (Bosinger et al., 2004).

Finally, numerous transcriptome studies explored differential gene expression in HIV-infected individuals. A first report in 2005 claimed to have found (Ockenhouse et al., 2005) a 10-gene signature for HIV-1 serostatus and a 6-gene signature for subjects experiencing a CD4+ T-cell decrease (Ockenhouse et al., 2005). The genes identified were primarily linked with immune response and apoptosis, mitochondrial function, and RNA binding (downregulated in subjects with better prognosis) (Ockenhouse et al., 2005). A study focusing on HIV-1–resistant individuals (Huang et al., 2011) found a set of 185 HIV-1 resistance genes, suggesting a major role for *nef* in disease pathogenesis, and among them pointed out 29 potential targets for AIDS prevention or therapy (Huang et al., 2011). By comparing the complementary DNA (cDNA) profiles of CD3+ T-cells in long-term non-progressors vs. medium progressors (Salgado et al., 2011), 325 genes appeared over-expressed in regular progressors (from DNA replication, cell cycle, and DNA damage pathways), vs. 136 over-expressed genes in long-term non-progressors (from cytokine–cytokine receptor interaction and negative control of apoptosis pathways) (Salgado et al., 2011). The transcriptome comparison of CD4+ T-cells and CD8+ T-cells from rapid progressors, viremic non-progressors, and elite controllers showed a lower expression of IFN-stimulated genes and an upregulation of *CASP1*, *CD38*, *LAG3*, *TNFSF13B*, *SOCS1*, and *EEF1D* genes in viremic non-progressors (Rotger et al., 2011). Finally, a transcriptomic screening also targeted miRNA expression profiles in peripheral blood mononuclear cell (PBMC) from rapid and chronic progressors and identified five downregulated miRNAs in rapid progressors that all converged to the apoptosis pathway (Zhang et al., 2013).

## Proteomic and Epigenomic Studies

Some proteomic studies have also been performed, but they were not very reproducible, as indicated in a recent review by Donnelly and Ciborowski (2016). To our knowledge, few epigenomic studies have been published to date in HIV/AIDS. One Korean group performed two chromatin immunoprecipitation sequencing (ChIP-seq) analyses in HIV latently infected CD4 T-cell lines to investigate the impact of H3K4me3 and H3K9ac histone modifications on latency. They revealed several potential candidate genes, including *NFIX*, tumor necrosis factor (TNF) receptor association factor 4 (*TRAF4*), and cell cycle regulating genes such as *CDKN1A* (p21) and *CCND2* (Park et al., 2014; Kim et al., 2017). Finally, the blood DNA methylation signatures of HIV-infected and uninfected subjects were compared through an epigenome-wide association study (EWAS), which highlighted a down-methylation of *NLC5* promoter in HIV-infected subjects (Zhang et al., 2016). This host gene encodes a key regulator of class I *HLA* gene expression and confirms the major role of the *MHC* locus in HIV viral control. Interestingly, *NLC5* promoter and additional *MHC* clusters also appeared differentially methylated in HIV–Hepatitis C virus (HCV) co-infected subjects (Zhang et al., 2017), emphasizing the importance of inflammation-related genes in the course of HIV infection. Overall, these studies are promising and underline the need for additional large-scale epigenetic studies in order to better capture the breadth of host–HIV complex interactions.

## Conclusion and Future Directions

In this review, we have presented numerous large-scale genomic and transcriptomic analyses that have taken place in the AIDS field, which are the consequences of the progress in molecular biology and biochemistry technologies. One can see that a huge research effort has been dedicated to genetic association studies, and this is logical since this experimental approach deals with real *invivo* data, i.e., cohorts of patients and HIV-1 infection *invivo*. Nevertheless, it was slightly surprising to observe that the main signals found by GWAS, in the *HLA* and *CCR5* loci, had already been identified by previous candidate gene approaches. This apparent limitation could be explained by the yet-unidentified role of other polymorphisms such as copy number variations (CNVs) or interacting gene variants. It could also be explained by the statistical constraints (such as stringent multiple testing corrections) that limit the use of genetic association data (numerous false negatives) and the overall low number of samples at stake (a few thousands) compared to other human diseases such as diabetes or obesity (hundreds of thousands) (Shungin et al., 2015; Fuchsberger et al., 2016). In light of the available biological information provided by the other large-scale studies such as transcriptomic or functional genomic studies presented in this review, it appears important to reanalyze the genomic data by integrating biological information in order to enhance the genetic association results. For instance, our group has successfully implemented such approaches by pre-selecting relevant SNPs defined either by their low frequency (Le Clerc et al., 2011) or by their functional impact as potential expression quantitative trait loci (eQTLs) (Spadoni et al., 2015). More generally, there are several methods for data integration, the first one being to cross-check the results obtained by one method through another, for instance, using GWAS to identify SNPs with low P-values, even non-significant, and then using transcriptomics to pick genes that are differentially expressed in a relevant cell type or tissue. By combining two (or more) methods, researchers can zoom in on specific genes of high interest. This has been implemented with the development of PrediScan (Gamazon et al., 2015). Another example of cross-checking is the results obtained by metabolome analysis and GWAS in which the researchers have found that metabolites present at high levels in the blood of some subjects are highly correlated with specific variants present in the genes of enzymes involved in their metabolism (Illig et al., 2010). Other methods of data integration rely on rescuing genes by correlating signals not only at the gene level but also at the pathway level: for instance, one can suspect that if a gene X in a biological pathway is important for a clinical phenotype, the genes present upstream in the biological pathway may impact this gene X expression and, as a consequence, also become targets of interest. One will thus have to look for cross-checks at the level of pathways (Chen et al., 2011). Importantly, it is essential for data integration to perform all these cross-checks in a smart and automated manner. Finally, more sophisticated statistical approaches have recently emerged outside of the HIV field, such as the Bayesian method for data integration (Kichaev et al., 2014; Pickrell, 2014; Finucane et al., 2015; Yang et al., 2017; International Multiple Sclerosis Genetics Consortium, 2019). These new methods are yet to be implemented in the relatively small HIV/AIDS cohorts but might reveal novel underlying physiopathological mechanisms.

With the massive research effort to fight AIDS, this has been a true field of experimentation and development for novel technologies. A first challenge is now the cross-usage of all this information gathered from so many large-scale studies, to transform this “gold mine” into diagnostic or therapy strategies to fight AIDS, and the same integration of omics big data should of course take place also for other human diseases. This systems biology challenge has not yet been met. A second challenge is to pursue the exploration of alternative technologies such as epigenomics or proteomics to derive more understanding of HIV-1 molecular pathogenesis. We hope that the AIDS field will remain a “cultural” leader for research progress in order to fully understand the molecular mechanisms at stake in HIV-1 infection and AIDS and allow the rationale development of diagnostic and therapeutic strategies to finally tackle the HIV-1 virus.

## Author Contributions

SLC, SL, and J-FZ conceived this review, performed the bibliography search, and wrote it in a collective manner.

## Funding

The authors have had their work funded from several sources. This review was directly funded by their employer: Conservatoire National des Arts et Métiers et Ecole Centrale de Nantes.

## Conflict of Interest Statement

The authors declare that the research was conducted in the absence of any commercial or financial relationships that could be construed as a potential conflict of interest.
